# Diversity of rhizosphere microbial communities in different rice varieties and their diverse adaptive responses to saline and alkaline stress

**DOI:** 10.3389/fmicb.2025.1537846

**Published:** 2025-04-08

**Authors:** Yu Zhong, Hai Chi, Tao Wu, Wenbo Fan, Haoyu Su, Ruoyu Li, Wenzhu Jiang, Xinglin Du, Ziming Ma

**Affiliations:** ^1^Jilin Provincial Engineering Laboratory of Plant Genetic Improvement, College of Plant Science, Jilin University, Changchun, China; ^2^Key Laboratory of Inland Saline-Alkaline Aquaculture, Ministry of Agriculture and Rural Affairs, East China Sea Fisheries Research Institute, Chinese Academy of Fishery Sciences, Shanghai, China; ^3^Max-Planck-Institute of Molecular Plant Physiology, Potsdam-Golm, Germany

**Keywords:** saline stress, alkaline stress, rice, rhizosphere microbiota, metagenomic analysis

## Abstract

Rice rhizosphere microbiota plays a crucial role in crop yield and abiotic stress tolerance. However, little is known about how the composition and function of rhizosphere soil microbial communities respond to soil salinity, alkalinity, and rice variety in rice paddy ecosystems. In this study, we analyzed the composition and function of rhizosphere soil microbial communities associated with two rice varieties (Jida177 and Tongxi933) cultivated in soils with different levels of salinity-alkalinity in Northeast China using a metagenomics approach. Our results indicate that the rhizospheres of Jida177 and Tongxi933 rice varieties harbor distinct microbial communities, and these microbial communities are differentiated based on both soil salinity-alkalinity and rice varieties. Furthermore, the observed differences in rice yield and grain quality between the Jida177 and Tongxi933 rice varieties suggest that these changes may be attributed to alterations in the rhizosphere microbiome under varying salinity conditions. These findings may pave the way for more efficient soil management and deeper understanding of the potential effects of soil salinization on the rice rhizosphere system.

## 1 Introduction

Rice (*Oryza sativa* L.) is a critical staple crop in the world, playing a vital role in maintaining food security. According to the latest data from the Food and Agriculture Organization of the United Nations (FAO), approximately 331 million hectares of land worldwide are affected by varying degrees of salinization, which constitutes 6.5% of the total land area. In China, saline soils cover 99 million hectares, with 80% of these saline resources remaining underutilized (Wang et al., 2011; Nan et al., 2022).

China is the largest producer of rice, contributing over 37% of global rice production in 2020. Recently, rice-based agricultural systems have been increasingly employed for the reclamation and utilization of saline soils. Rice yield and grain quality are crucial indicators for evaluating the effectiveness of saline land improvement (Murtaza et al., 2009; Huang et al., 2021; Ma and Hu, 2023; Ma et al., 2024; Ma and Hu, 2024). Despite numerous studies reporting improvements in saline soils through rice cultivation, the underlying mechanisms remain poorly understood.

Microorganisms are highly diverse and inhabit various environments, forming intricate communities that play a crucial role in biogeochemical cycles, significantly influencing soil health, quality, and fertility (Xue et al., 2018; Hou et al., 2020). Among the plant-associated microbiomes, the rhizosphere is a key area of focus due to its rich microbial diversity and its role in essential ecological processes. The rhizosphere, a zone of interaction between plants and soil, is actively involved in organic matter decomposition, nutrient cycling, and other microbe-driven processes. Plant roots influence the microbial community by releasing exudates such as carbohydrates, amino acids, and other secondary metabolites, which attract a range of microorganisms. Functional groups of these microorganisms include phosphate solubilizers, iron reducers, nitrogen fixers, methane cyclers, and fermenters (Breidenbach et al., 2016; Ding et al., 2019). It is proposed that plants selectively modulate the rhizosphere microbiome, promoting the growth of beneficial microbes that enhance plant health and growth. In addition, plant genotype plays an integral role in shaping the root microbiome (Dhondge et al., 2022), with genotypic differences affecting the microbiome composition, particularly in annual crops, due to variations in plant physiology.

Despite the importance of understanding these interactions, the overall impact of rice cultivation on the ecosystem functions of saline soils, particularly in terms of nutrient cycles, remains insufficiently studied. Research on this area is critical for improving food supply and advancing global agricultural sustainability (Toju et al., 2018). A thorough understanding of the taxonomic and functional composition of the rhizosphere microbiome is crucial for developing strategies to enhance plant performance and reduce anthropogenic impacts, thereby promoting sustainable ecosystem functions (Ling et al., 2022). Although previous studies have explored the rice rhizosphere (Bhattacharyya et al., 2016; Sessitsch et al., 2012), the overall patterns of genomic and functional composition, particularly in relation to nutrient cycling, across different soil salinity levels and rice varieties have yet to be fully investigated. Metagenomic approaches provide a valuable tool for uncovering the taxonomic abundance and functional gene profiles within the rice rhizosphere microbiome, offering deeper insights into microbial diversity and their functional roles (Quince et al., 2017; Bhattacharyya et al., 2017).

In this study, we employed metagenomic techniques to investigate how the composition and functionality of rhizosphere soil microbial communities are influenced by soil salinity-alkalinity and rice varieties in rice field ecosystems in northeast China. Specifically, we aimed to address two key questions: (1) How do soil salinity-alkalinity and rice varieties impact the taxonomic composition of the rice rhizosphere microbiome? (2) How do these factors affect the functional structure of the microbial community?

## 2 Materials and methods

### 2.1 Analysis of rice yield and grain quality

Rice yield was determined by panicle number, grains per panicle, 1,000-grain weight (TGW), and seed setting rate. These traits were photographed and investigated using a Nikon digital camera and scanner. Seed length and width were measured for at least 20 mature seeds using vernier calipers. Grain quality was assessed using a grain analyzer, following the method described by Wu et al. (2014).

### 2.2 Soil sampling regime

A total of 12 samples (2 varieties x 2 fields x 3 replicates) were collected from rice fields located in Jilin Province (45°3′5.64840″N, 123°13′19.801200″E) in October 2023. Two rice varieties Jida177 (J177) and Tongxi933 (T933) were used in this study. Two rice fields were selected, each with distinct soil pH values (pH 7.56 and pH 8.20) and salinity (1.92 g/kg and 2.99 g/kg). The field with low pH and salinity was regarded as low saline-alkaline soil, the other one with high pH and salinity was regarded as high saline-alkaline soil. The soil sample was collected during the rice maturity stage. Soil samples from low saline-alkaline soil and high saline-alkaline soil planted J177 were designated as LJ177 and HJ177, respectively. Similarly, soil samples from fields planted T933 were named as LT933 and HT933, respectively.

For each replicate, five rice individuals obtained from designated plots within a 2 × 2 m area. These individuals were collected from the corners and center of the designated plot within a single rice field, in accordance with the sampling protocol established by Guo et al. (2022). To minimize disturbance to the rhizosphere, rice plants were carefully extracted using a gardening fork and shovel. The loosely bound soil surrounding the rhizosphere was gently shaken off, and rice plants along with their root systems were placed in sterile plastic bags. These samples were subsequently transported to the laboratory in an icebox to maintain optimal conditions. In the laboratory, soil tightly adhering to the roots was carefully brushed off, following the method described by Guo et al. (2022). The rhizosphere soil from five individual plants within one plot, serving as a replicate, was pooled and thoroughly mixed to ensure uniformity. One portion of this rhizosphere soil was immediately stored at −80°C for DNA extraction and sequencing. The remaining sample was utilized for the measurement of various soil parameters, including pH, salinity, soil organic carbon (SOC), total nitrogen (TN), soil alkaline hydrolysis nitrogen (AN), available phosphorus (AP), and available potassium (AK) (Olsen, 1954; Bremner, 1960; Mebius, 1960; Page et al., 1982).

In addition, we also collected soil samples from two distinct rice fields before rice planting. Soil samples collected from low saline-alkaline soil and high saline-alkaline soil were designated as LCK and HCK, respectively. Soil sampling method is the same as described above. These samples were also investigated the physiochemical properties.

### 2.3 Measurement of soil physical and chemical properties

Soil pH was investigated using a pH meter with a soil-water ratio of 1:2.5. Salinity was measured using a salinity meter with a soil-water ratio of 1:5. Soil organic carbon (SOC) content was investigated using potassium dichromate oxidation (Mebius, 1960). Total nitrogen (TN) content was determined using the Kjeldahl method (Bremner, 1960). Soil alkaline hydrolysis nitrogen (AN), available phosphorus (AP) and available potassium (AK) were measured according to Soil Agrochemical Analysis Method (Olsen, 1954; Page et al., 1982).

### 2.4 DNA extraction, library construction, and metagenomic sequencing

Total genomic DNA was extracted from soil samples using the Mag-Bind® Soil DNA Kit (Omega Bio-tek, Norcross, GA, U.S.) according to manufacturer’s instructions. The concentration and purity of extracted DNA was determined with TBS-380 and NanoDrop2000, respectively. The quality of the DNA extract was checked on a 1% agarose gel. The DNA extract was fragmented to an average size of about 400 bp using the Covaris M220 (Gene Company Limited, China) for paired-end library construction. A paired-end library was constructed using NEXTFLEX Rapid DNA-Seq (Bioo Scientific, Austin, TX, USA). Adapters containing the full complement of sequencing primer hybridization sites were ligated to the blunt-end of fragments. Paired-end sequencing was performed on an Illumina NovaSeq (Illumina Inc., San Diego, CA, USA) using NovaSeq 6,000 S4 Reagent Kit v1.5 following the manufacturer’s instructions.^1^ Sequence data generated in this project have been deposited in the NCBI Short Read Archive database (SRA Number: xxxx).

### 2.5 Sequence quality control and genome assembly

The raw reads were trimmed of adaptors, and low-quality reads (length < 50 bp or with a quality value <20 or having N bases) were removed by fastp v0.20.0 (Chen et al., 2021). Clean reads after the quality control were assembled using MEGAHIT v1.1.2 (Li et al., 2015). Contigs with a length ≥ 300 bp were chosen as the final assembling result and used for following gene prediction and annotation.

### 2.6 Gene prediction, taxonomy, and functional annotation

Open reading frames (ORFs) from each assembled contigs were predicted using Prodigal. The predicted ORFs with a length ≥ 100 bp were retrieved and translated into amino acid sequences using the NCBI translation table.^2^ A non-redundant gene catalog was constructed using CD-HIT v4.6.1 (Fu et al., 2012) with 90% sequence identity and 90% coverage. Gene abundance of non-redundant genes was estimated for each sample by SOAPaligner v2.21 with 95% identity (Gu et al., 2013).

The non-redundant gene catalog was aligned against the NCBI NR database using DIAMOND with an e-value of 1e-5 (Buchfink et al., 2015). Reference protein IDs of best hits were deployed to disentangle the taxonomic affiliation. The functional annotation was also performed for the non-redundant gene catalog. The non-redundant genes were aligned to Kyoto Encyclopedia of Genes and Genomes (KEGG) database (Kanehisa and Goto, 2000) and the Carbohydrate-Active enZymes (CAZy) database (Drula et al., 2022) using DIAMOND with an e value of 1e-5 (Buchfink et al., 2015).

### 2.7 Statistical analyses

All statistical analysis were performed using R software (R Core Team, 2018). Significant differences among groups were estimated by duncan tests using R package “agricolae” (De Mendiburu, 2023). Pairwise correlation analysis between soil characteristics and composition of microbial community were calculated by mantel test using R package “ape” (Paradis and Schliep, 2019).

## 3 Results

### 3.1 Effect of saline-alkaline stress on rice yield and grain quality

**Figure 1:**
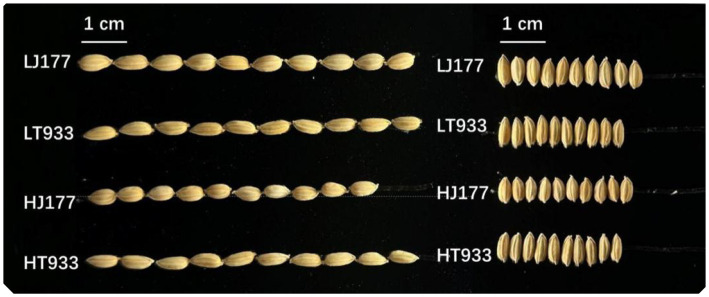
Grain morphology. LJ177, low saline-alkaline soil Jida177; LT933, low saline-alkaline soil Tongxi933; HJ177, high soil saline-alkaline Jida177; HT9333, high soil saline-alkaline Tongxi933.

In the investigation of rice yield, saline-alkaline soil was found to influence key parameters such as panicle number, grains per panicle, seed setting rate, and TGW, with significant differences observed between two rice varieties (Table 1). In the field with high saline-alkaline soil (pH 8.20 and salinity 2.99 k/kg), the panicle number of Jida177 and Tongxi933 increased by 15 and 4%, respectively, while grains per panicle increased by 7% for Jida177 and 9% for Tongxi933 in the field with low saline-alkaline soil (pH 7.56 and Salinity 1.92 k/kg). The seed setting rate was not affected by soil conditions for both Jida177 and Tongxi933. High saline-alkaline soil resulted in a reduction in TGW for Jida177, while no significant change was observed for Tongxi933. Jida177 showed better tolerance to high saline-alkaline soil than Tongxi933, as evidenced by its higher total grain number. TGW showed more differences between two rice varieties, while salinity-alkalinity affected TGW less for two rice varieties. Although a decrease in TGW was observed for Jida177, its total yield was still higher than that of Tongxi933. In high saline-alkaline soil, seed length and width of Jida177 decreased by 9 and 6%, respectively, while no significant changes were observed in Tongxi933 (Figure 1).

**Table 1:** Effect of soil saline-alkaline stress on rice yield and grain quality.

	LJ177	LT933	HJ177	HT933
Panicle number	13.50 ± 1.29b	17.25 ± 2.75a	15.50 ± 1.00ab	18 ± 1.41a
Grains per panicle	180.42 ± 1.4a	136.58 ± 7.07b	192.03 ± 20.59a	148.75 ± 14.56b
Seed setting rate	0.92 ± 0.01a	0.94 ± 0.04a	0.93 ± 0.02a	0.93 ± 0.02a
1,000-grain weight (g)	25.05 ± 1.70a	20.18 ± 0.37c	22.59 ± 0.56b	21.39 ± 0.39bc
Seed length (mm)	7.21 ± 0.05c	7.37 ± 0.06b	6.55 ± 0.03d	7.56 ± 0.03a
Seed width (mm)	3.71 ± 0.04a	3.02 ± 0.02c	3.48 ± 0.05b	3.07 ± 0.01c
Protein (%)	6.81 ± 0.65bc	6.43 ± 0.39c	8.26 ± 0.73a	7.68 ± 0.84ab
Fat (%)	2.01 ± 0.21a	2.41 ± 0.40a	2.16 ± 0.59a	2.54 ± 0.43a
Amylose (% total starch)	18.04 ± 0.05a	17.99 ± 0.18a	17.79 ± 0.13b	18.11 ± 0.16a

Different letters in the same line represent significant differences at *p* < 0.05 according to Duncan test.

The protein, fat, and amylose contents primarily determined the nutritional quality of the grain. We also found that the protein and amylose contents of rice seeds were impacted by soil salinity and alkalinity, while the fat content was not affected (Table 1). In high saline-alkaline soil, the protein content of Jida177 and Tongxi933 increased by 21.3 and 19.4%, respectively, while the amylose content of Jida177 decreased by 1% and that of Tongxi933 remained unchanged (Table 1).

### 3.2 Effect of rice cultivation on soil physicochemical properties

Rice cultivation has significant impact on soil physicochemical properties including pH, salinity, soil organic carbon (SOC), soil total nitrogen (TN), soil alkaline hydrolysis nitrogen (AN), soil available phosphorus (AP) and soil available potassium (AK) content (Table 2). After rice cultivation, soil pH and salinity decreased in two rice fields after rice cultivation. However, SOC, TN, AN, AP and AK showed diametrically opposite trends.

**Table 2:** Physical and chemical characteristics of rhizosphere soils of rice cultivated in saline-alkali rice fields.

Properties	LCK	LJ177	LT933	HCK	HJ177	HT933
pH	7.56 ± 0.05b	6.58 ± 0.08e	6.96 ± 0.06d	8.2 ± 0.02a	6.96 ± 0.05d	7.17 ± 0.1c
Salinity (g kg^−1^)	1.92 ± 0.04b	0.78 ± 0.01d	0.84 ± 0.04d	2.99 ± 0.08a	0.79 ± 0.05d	1.16 ± 0.07c
SOC (g kg^−1^)	17.58 ± 0.75d	29.63 ± 0.97a	28.23 ± 1.31b	14.2 ± 0.57e	28.34 ± 1.04b	22.14 ± 0.77c
TN (g kg^−1^)	0.75 ± 0.02c	0.92 ± 0.01a	0.89 ± 0.01b	0.56 ± 0.02f	0.63 ± 0.01d	0.6 ± 0.01e
AN (mg kg^−1^)	70.94 ± 0.97d	95.91 ± 2.7a	84.6 ± 3.16c	67.42 ± 1.04e	90.17 ± 3.08b	72.17 ± 3.02d
AP (mg kg^−1^)	41.33 ± 0.49d	54.92 ± 1.84a	48.76 ± 0.96c	37.25 ± 0.94e	51.63 ± 1.38b	43.55 ± 1.71d
AK (mg kg^−1^)	172.24 ± 2.33d	203.41 ± 2.65b	188.07 ± 1.89c	171.14 ± 1.95d	208.86 ± 2.34a	189.92 ± 1.12c

SOC, soil organic carbon; TN, soil total nitrogen; AN, soil alkaline hydrolysis nitrogen; AP, soil available phosphorus; AK, soil available potassium. Different letters in the same line represent significant differences at *p* < 0.05 according to Duncan test.

In the rice field with low saline-alkaline soil, soil pH and salinity decreased by 13 and 59% after planting Jida177, and these decreased by 8 and 56% after planting Tongxi933 (Table 2). However, soil pH and salinity decreased by 15 and 73% after planting Jida177 and by 15 and 73% after planting Tongxi933 in the rice field with high saline-alkaline soil. The SOC contents in two rice fields were significantly increased after rice cultivation. However, the AN, AP and AK contents changed little after rice cultivation (Table 2).

### 3.3 Effect of rice cultivation on soil microbial diversity

We compared the community diversity of rhizosphere microbiomes among LJ177, HJ177, LT933, and HT933. There was no significant difference in the Chao1 richness of the rhizosphere communities among four samples (Table 3). This indicated that the rhizosphere microbiome of four samples possessed similar community diversity, regardless of the saline-alkaline level. Shannon’s diversity index and Simpson’s index showed that high saline-alkaline soils harbored more diverse bacterial and fungal taxa than low saline-alkaline soils, with HJ177 exhibiting the most diversity (Table 3).

**Table 3:** Alpha diversity analysis within the rhizosphere community of rice varieties under study.

	LJ177	LT933	HJ177	HT933
Chao	22,800 ± 99a	22,662 ± 75a	22,673 ± 102a	22,860 ± 170a
Shannon	5.98 ± 0.02b	5.98 ± 0.02b	6.06 ± 0.01a	6.04 ± 0.01a
Simpon	0.012 ± 0.00a	0.012 ± 0.00a	0.010 ± 0.00b	0.011 ± 0.01b
Coverage	1	1	1	1

Different letters in the same line represent significant differences at *p* < 0.05 according to Duncan test.

**Figure 2:**
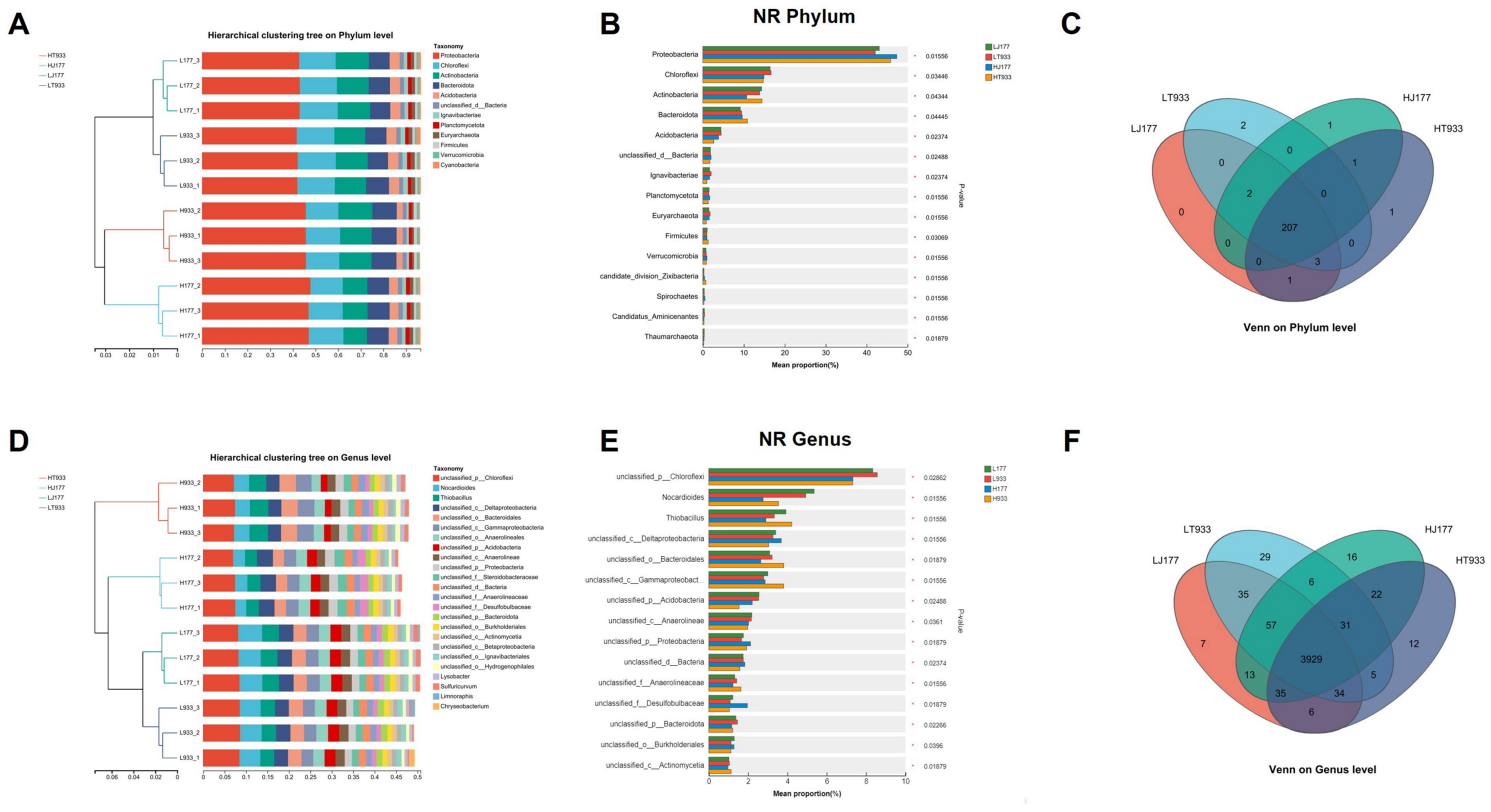
**(A)** The microbial community composition (phylum level) in saline-alkaline rice fields. **(B)** The relative abundance of the top 15 phylum in saline-alkaline rice fields. **(C)** Venn diagram of phylum distribution in saline-alkaline rice fields. **(D)** The microbial community composition (genus level) in saline-alkaline rice fields. **(E)** The relative abundance of the top 15 phylum in saline-alkaline rice fields. **(F)** Venn diagram of genus distribution in saline-alkaline rice fields. LJ177, Jida177 grown in low saline-alkaline soil; LT933, Tongxi933 grown in low saline-alkaline soil; HJ177, Jida177 grown in high saline-alkaline soil; HT9333, Tongxi933 grown in high saline-alkaline soil.

The taxonomic profiling of the metagenome was carried out at phylum and genus levels. The community composition of LJ177 and HJ177 were distinguished from that of LT933 and HT933 at both of levels (Figures 2A,D). This indicated that the rhizosphere microbiomes were different not only across different saline-alkaline soil types, but also between two rice varieties.

The abundance of the top 15 phyla in each sample was compared. The phylum Proteobacteria was found to be most abundant in LJ177 (43.04%), LT933 (42.06%), HJ177 (47.31%), and HT933 (45.84%), with a higher abundance in high saline-alkaline soils than in low saline-alkaline soils. Followed by Chloroflexi LJ177 (16.44%), LT933 (16.65%), HJ177 (14.94%), and HT933 (14.74%), with higher abundance in low saline-alkaline soils than in high saline-alkaline soils. The third most abundant phylum was Actinobacteria in LJ177 (14.32%), LT933 (13.84%), HJ177 (10.70%), and HT933 (14.40%) (Figure 2B). In terms of taxonomic composition, the relative abundance of the phyla Chloroflexi and Acidobacteria decreased significantly in HJ177 and HT933 compared with that in LJ177 and LT933, while the relative abundance of Proteobacteria and Actinobacteria increased significantly among comparisons (Figure 2B). However, the relative abundance of Actinobacteria decreased significantly in HJ177 compared with LJ177, while there is no significant change between LT933 and HT933. The relative abundance of the genera unclassified_p_Chloroflexi, Nocardioides, and unclassified_p_Acidobacteria decreased significantly in HJ177 and HT933 compared with that in LJ177 and LT933, while the relative abundance of Thiobacillus and unclassified_p_Bacteroidales increased significantly between LT933 and HT933, in contract, decreased significantly between LJ177 and HJ177 (Figure 2E). There are 215 phyla detected in LJ177 and LT933. Only one phylum, namely p_Candidatus_Harrisonbacteria, was exclusively belonged to LJ177 and two phyla namely p_Foraminifera and p_Preplasmiviricota were specific to LT933. Remaining 212 phyla were shared between LJ177 and LT933 (Figure 2C). A total of 216 phyla were detected in HJ177 and HT933. Of them, 208 phyla were shared between HJ177 and HT933. Only three phyla, namely p_Phixviricota, p_Euglenozoa and p_Cressdnaviricota, were exclusively belonged to HJ177, and remaining five phyla (p_Candidatus_Terrybacteria, p_Candidatus_Tagabacteria, p_Evosea, p_Candidatus_Hydrothermarchaeota, and p_Candidatus_Spechtbacteria were specific to HT933) (Figure 2C). In category of genera, the percentage abundance of the top 15 genera in each sample was compared. The genus unclassified_p__Chloroflexi was found to be most abundant in LJ177 (8.34%), LT933 (8.57%), HJ177 (7.33%), and HT933 (7.31%), with higher abundance in low saline-alkaline soils than in high saline-alkaline soils. Nocardioides LJ177 (5.35%), LT933 (4.92%), HJ177 (2.76%), and HT933 (3.54%) were found to be more abundant in low saline-alkaline soils than in high saline-alkaline soils. Thiobacillus LJ177 (3.92%), LH933 (3.33%), HJ177 (2.90%), and HT933 (4.22%) were the third most abundant phylum (Figure 2D). A total of 4,187 genera were detected in LJ177 and LT933. The specific genera found in LJ177 and LT933 were 61 and 71, respectively. The most of genera (4055) were common to LJ177 and LT933 (Figure 2E). In HJ177 and HT933, a total of 4,166 genera were detected. Of them, 92 genera were exclusively associated with HJ177, and 57 genera were specific to HT933. Remaining 4,017 genera were shared between HJ177 and HT933 (Figure 2F).

### 3.4 The response of microbial community composition and functional structure in relation to soil saline-alkaline

**Figure 3:**
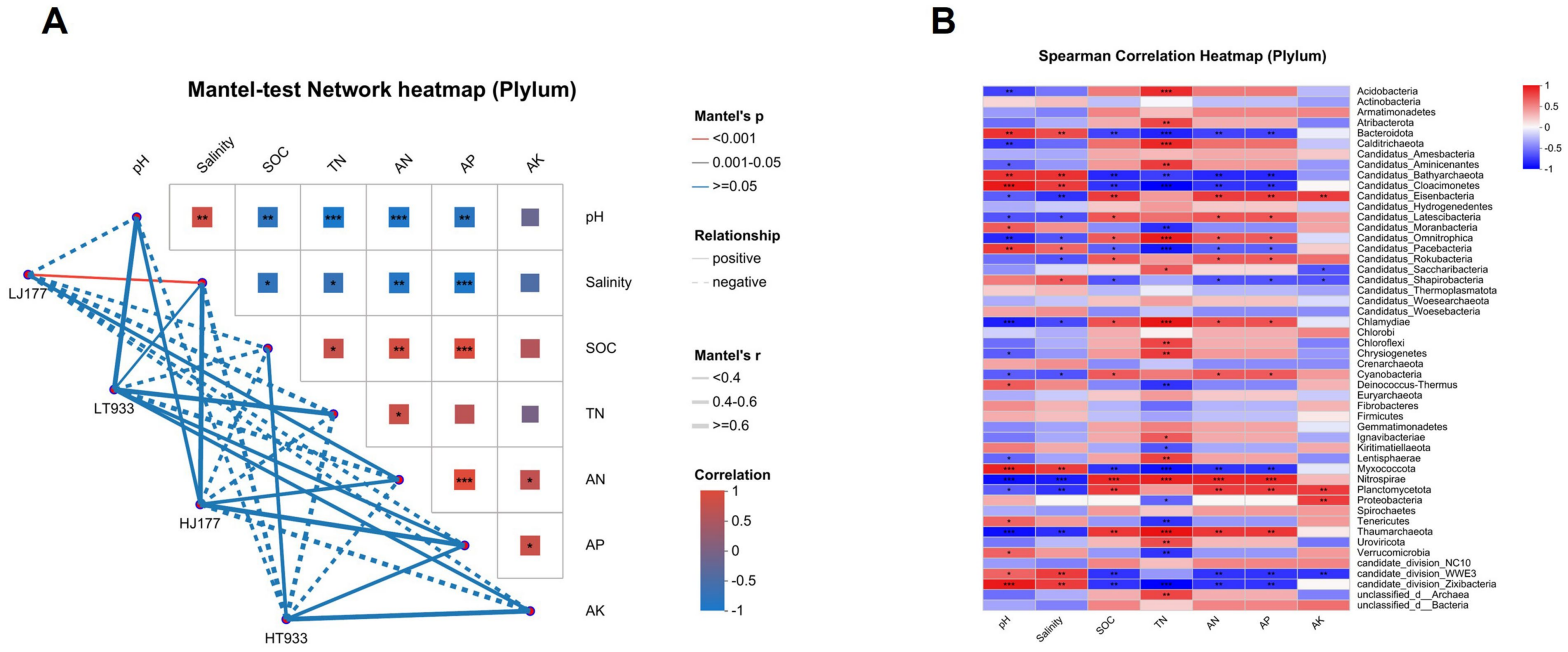
**(A)** Pairwise correlation analysis between soil characteristics and composition of microbial community in saline-alkaline rice fields based on the mantel tests. Color gradient and block size denote Pearson’s correlation coefficients. The color of the line represents the significance of the differences (*p* values) based on 999 permutations. The size of the line represents correlation coefficients (Mantel’s R). Asterisks in the block denote for different significance levels at *p < 0.05, **p < 0.01 and ***p < 0.001. **(B)** Correlation of species with environmental factors. Asterisks denote for different significance levels at *p < 0.05, **p < 0.01 and ***p < 0.001.

We found that soil physicochemical properties SOC, TN, AN, and AP were negatively correlated with soil pH and salinity. AK showed no significant correlation with soil pH and salinity (Figure 3). Taxonomic and functional gene composition of rhizosphere microbes were significantly affected by soil pH and salinity (Figure 3). The relative abundance of seven phyla presented a positive and significant correlation with soil pH and salinity, while their relative abundances were negatively and significantly correlated with SOC, TN, AN, and AP (Figure 3). These phyla included Bacteroidota, Candidatus_Bathyarchaeota, Candidatus_Cloacimonetes, Candidatus_Omnitrophica, Candidatus_Pacebacteria, Myxococcota, candidate_division_WWE3, candidate_division_Zixibacteria. Meanwhile, only Atribacterota, Calditrichaeota,Chloroflexi, Uroviricota, and unclassified_d_Bacteria were positively and significantly correlated with soil TN.

**Figure 4:**
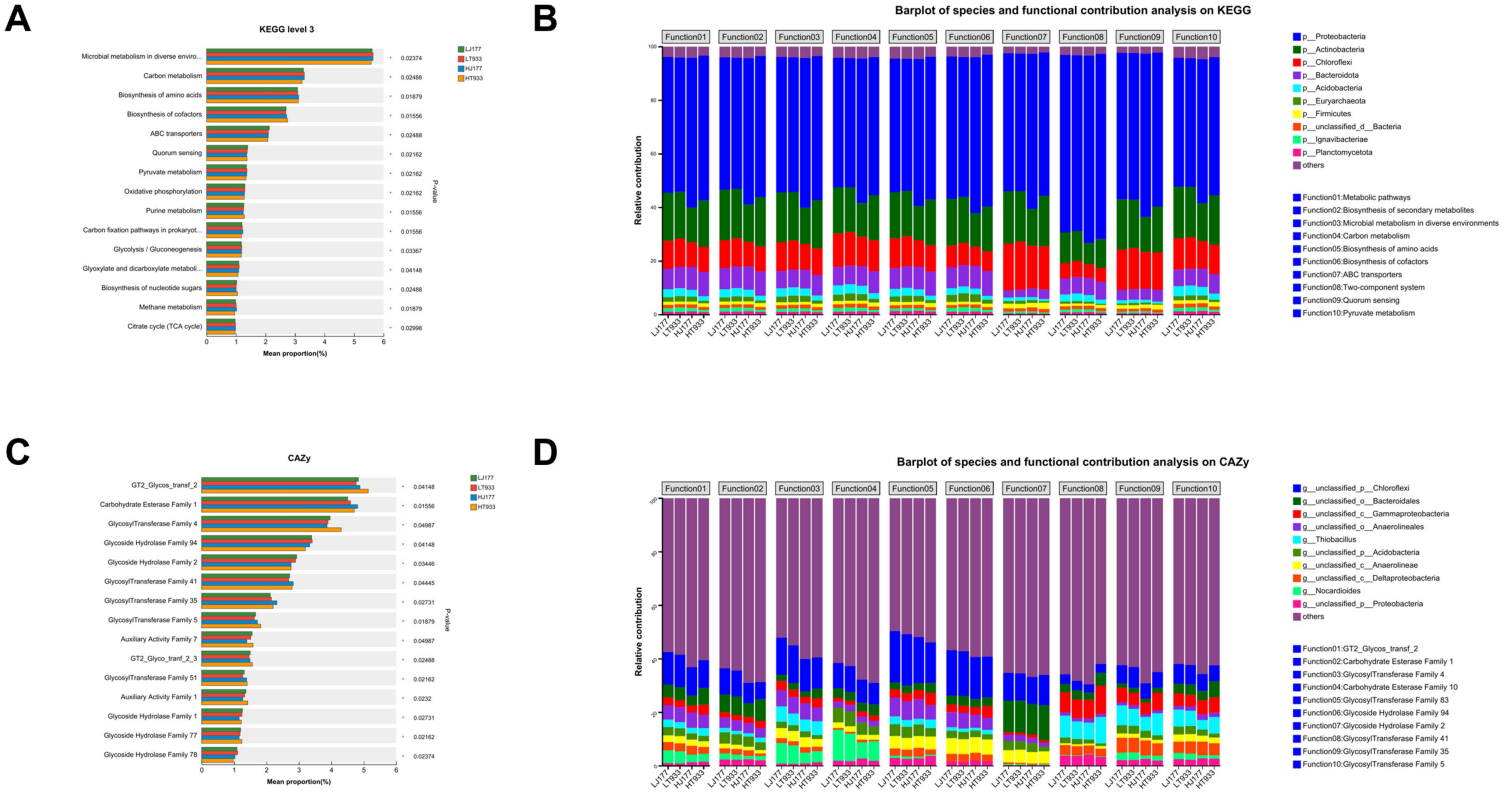
**(A)** The relative abundance of the top KEGG level 3 in saline-alkaline rice fields. **(B)** The species and functional contribution analysis on KEGG in saline-alkaline rice fields. **(C)** The relative abundance of the top 15 CAZy in saline-alkaline rice fields. **(D)** The species and functional contribution analysis on CAZy in saline-alkaline rice fields. LJ177, Jida177 grown in low saline-alkaline soil; LT933, Tongxi933 grown in low saline-alkaline soil; HJ177, Jida177 grown in high saline-alkaline soil; HT9333, Tongxi933 grown in high saline-alkaline soil.

The relative abundances of functional genes at KEGG pathway were compared among LJ177, HJ177, LT933, and HT933. We obtained the top15 functional pathways with significant differences at four samples, including “microbial metabolism in diverse environments,” “carbon metabolism,” “biosynthesis of amino acids,” “biosynthesis of cofactors,” and “ABC transporters” (Figure 4A). In the differential KO functional categories, the relative contribution of microbial community composition is different (Figure 4B). The relative contribution of p_Proteobacteria was dominant in all top 10 functional categories. Of them, the relative contribution of p_Proteobacteria was the most in KO function involved in “Two-component system.” These 10 KO functional categories were involved in “Metabolic pathways,” “Biosynthesis of secondary metabolites,” “Microbial metabolism in diverse environments,” “Carbon metabolism,” “Biosynthesis of amino acids,” “Biosynthesis of cofactors,” “ABC transporters,” “Two-component system,” “Quorum sensing,” and “Pyruvate metabolism.” In these KO functional categories, the relative contribution of p_Chloroflexi in the functional gene affiliated with “ABC transporter” was the most, while the minimal relative contribution of p_Bacteroidota was found in this KO function. We also investigated the relationship between the relative contribution of species and the functional genes for carbohydrate-active enzymes (CAZy) (Figures 4C,D). The dominantly and significantly different CAZy included “GT2_Glycos_transf_2,” “Carbohydrate Esterase family 1,” “Glycosyl Transferase Family 4,” “Glycoside Hydrolase Family 94” (Figure 4C). The main species participating in the functions listed above were g_unclassified_p_Chloroflexi, g_unclassified_o_Bacteroidales, g_unclassified_c_Gammaproteobacteria, g_unclassified_o_Anaerolineales and g_Thiobacillus (Figure 4D). Meanwhile, Nocardioides presented a special relative contribution in the functional categories involved in “Glycosyl transferase family 4” and “Carbohydrate esterase family 10” (Figure 4D). Together, these results indicated that both rhizosphere community composition and functional gene are significantly diverse among all samples.

## 4 Discussion

### 4.1 Effect of soil saline-alkaline stress on rice yield and grain quality

Soil salinity and alkalinity represents a major constraint to rice growth, adversely impacting both yield and quality. It significantly affects critical yield parameters, including seed setting rate, tiller number, panicle number, and panicle length (Shereen et al., 2005; Turan et al., 2007; Zhou et al., 2018).

In this study, the seed setting rate remained relatively stable under saline-alkaline conditions, while other yield traits exhibited considerable variation. Two rice varieties, Jida177 and Tongxi933, were grown in soils with high saline-alkaline levels. Jida177 showed a 15% increase in panicle number and a 6.3% increase in grains per panicle, whereas Tongxi933 exhibited a more modest 4% increase in panicle number and an 8.9% increase in grains per panicle. However, Jida177’s TGW decreased under the saline-alkaline condition, suggesting a possible association with decreased carbohydrate allocation to grain filling. Zeng and Shannon (2000) proposed a hypothesis that, under saline stress, a greater proportion of carbohydrates may be allocated to shoot growth during the post-anthesis phase in certain rice varieties, as opposed to being directed toward grain development. On the other hand, Tongxi933 was unaffected in terms of TGW under the same conditions.

Grain length is an important indicator of appearance quality, since longer grains are more likely to break down during the milling process, and rice planted in the saline soil showed a significantly decrease in the grain aspect ratio (Rao et al., 2013). Our findings indicated that rice grown in high saline-alkaline soil had shorter grains, resulting in a significantly reduction in grain aspect ratios. Specifically, Jida177 experienced a decrease in the grain length and width under these conditions.

Amylose and protein contents are essential indices of rice quality. Rice with low content of amylose is moist and sticky and has a good texture after cooked (Juliano, 1971). Protein is the second most important component after starch, and the higher the protein content, the better the rice quality (Chen et al., 2021). Compared with that of the normal condition, rice grown under salt stress had significantly lower amylose content, while the protein content increased (Zhou et al., 2018).

It has further been suggested that high salt stress increased the seed protein content, while low salt stress decreased the seed protein content (Sangwongchai et al., 2022). In our study, both Jida177 and Tongxi933 exhibited protein contents were increased under high saline-alkaline condition. Interestingly, amylose content was decreased in Jida177, while it was increased in Tongxi933. These trait differences also highlight the difference in saline-alkaline tolerance among rice varieties.

Higher yields in salt-tolerant cultivars can be attributed to differences in agronomic and physiological traits. As examples, greater photosynthetic rate, enlarged plant root system, elevated Na+/K+ ratio, higher proline content, better soluble carbohydrate content, and antioxidant enzyme activities have been observed in salt-tolerant cultivars (Li et al., 2023). These insights provide valuable information for breeding salt-tolerant rice varieties, enabling more effective utilization of saline-alkali soils.

### 4.2 Effect of rice cultivation on soil physicochemical properties

The findings of this study suggest that rice cultivation has a positive effect on soil physicochemical properties, particularly in terms of soil pH and salinity. During the cultivation period, both soil pH and salinity exhibited a gradual decrease, which aligns with the observations of Zhu et al. (2021). During the flooding phase of rice cultivation, the soil environment undergoes a shift from an oxidative state to a reductive state during the flooding phase, resulting in the consumption of alkaline ions, which subsequently reduces the soil pH value. In addition, the presence of water often dilutes the salt concentration in the soil in flooded fields. In this study, the soil salinity was decreased by 59 and 56% after planting Jida177 and Tongxi933 in the field with low saline-alkaline soil, respectively. Notably, the soil salinity was reduced more after planting Jida177 (73%) in the field with high saline-alkaline soil (Table 2), suggesting Jida177 exhibits a stronger tolerance to saline-alkaline stress compared to Tongxi933.

We found that rice cultivation improved SOC in saline soil, which was consistent with findings from Tan and Kang (2009) and Huang et al. (2012). The proportion of water-stable aggregates in paddy field soil was higher than that in saline-sodic wasteland (Feng et al., 2019), however, unstable soil aggregates caused by high sodium levels or erosion may heighten SOC exposure to microbial decomposition, leading to decreased SOC amounts (Wong et al., 2010). Moreover, the osmotic stress and poor soil structure in high-salt soils limit microbial activity, leading to reduce C turnover (Franklin et al., 2017). Rice cultivation mitigates soil salinity and alkalinity (Xu et al., 2020), which is beneficial for enhancing C levels in saline-alkaline soils. For instance, Xu et al. (2020) found that rice cultivation on coastal saline-alkaline soils increased the organic matter content by 44%. Similarly, Zhang et al. (2020) observed that 20 years of rice cultivation in saline-alkaline soil boosted the SOC content by 90.6%. This increase in SOC content may be attributed to long-term flooding of the paddy soil, in which the decomposition rate of SOC was slower than that of the dryland soil.

### 4.3 Effect of rice cultivation on soil microbial diversity

Previous studies reported on rice crops identified changes in the microbial community richness and diversity in the rhizosphere (Hussain et al., 2012). Imchen et al. (2019) also found variation in the richness and diversity of microbial communities in rice rhizosphere during various growth stages. Factors varying from soil-flood environment, temperature, pH, seasonal variations in different geo-climatic locations of rice fields, genotypes of host varieties, and their root exudates account for variations in the community structure (Okabe et al., 2000; Barreiros et al., 2011). In our study, the analysis of the composition of the overall rhizosphere microbial community revealed that the microbial associated with soil saline-alkali changed considerably in terms of richness and diversity. These differences may be caused by differences in soil saline-alkali at the sampling sites as well as differences in the genetic composition of rice varieties, both of which result in differences in root exudates that may be responsible for differences in species richness, diversity, and evenness. These factors have been identified as key influence shaping soil microbial communities.

The phylum-level taxonomic distribution indicated that the microbial community associated with the rhizosphere was composed of a number of taxa, among which the dominant taxon was proteobacteria. Proteobacteria are the largest and most metabolically diverse group of soil bacteria (Janssen, 2006). Arjun and Harikrishnan (2011) have identified Proteobacteria as the dominant phylum in rice. Apart from Proteobacteria, the most abundant taxa included Chloroflexi, Actinobacteria, Acidobacteria and Firmicutes (Chowdhury et al., 2009). Our findings are in line with these reports, the phylum Proteobacteria was found to be the most abundant in rice soil.

Previous studies have also revealed that environmental factors are the primary drivers shaping the organization of soil microbial communities (Philippot et al., 2013; Edwards et al., 2015). In addition to environmental factors, host genotype serves a key role in microbial associations (Wagner et al., 2016; Emmett et al., 2017). In our study, the proportion and abundance of unique microbial communities were lower than those of shared microbial communities at sampling sites with different soil salinity levels and rice varieties. Hence, our investigation may reveal that the soil saline-alkali content of different samples and rice varieties (Jida177 and Tongxi933) contribute critically to the formation of rhizobia communities.

The above results also suggest that, while genotype-induced differences in root exudates may also be responsible for the associated differences in the rhizosphere microbiota, the effect of the soil environment is much greater than genotypic differences in the same species.

Plants activate their stress defense systems in response to abiotic and biotic stress. Metabolites like salicylic acid (SA), jasmonic acid (JA), abscisic acid (ABA), indole-3-acetic acid (IAA), gibberellic acid (GA) and gamma-aminobutyric acid (GABA) regulate these responses and alter the rhizosphere microbiota (Kniskern et al., 2007; Carvalhais et al., 2013; Li et al., 2017). Accordingly, the defense mechanisms of Jida177 and Tongxi933 may differ, making Tongxi933 more susceptible to abiotic and biotic stresses. This may also explain the differences in the richness, diversity and composition of the microbiota associated with Jida177 and Tongxi933 varieties in the study.

### 4.4 The response of microbial community composition and functional structure in relation to soil saline-alkaline

Our results revealed that soil pH was the major driver of the rice rhizosphere microbial community composition and structure (Figure 3A). This result is consistent with the previous studies (Kuang et al., 2013). In our study, the bacterial phyla Chloroflexi and Acidobacteria were more abundant in low pH rhizosphere soils, whereas Proteobacteria were abundant in high pH rhizosphere soils (Figure 3B). Chloroflexi were well recognized for degrading polysaccharides in anoxic zones of rice field soils (Ahn et al., 2012). Acidobacteria thrived in well-drained cropland soils with low pH (Aguirre-von-Wobeser et al., 2018; Eichorst et al., 2018; Xu et al., 2022). Actinobacteria were involved in organic matter decomposition in oxic zones of rice paddies (Ahn et al., 2012) and were prevalent in higher pH and well-drained farmland soil (Wang et al., 2019). These were all enriched in rhizosphere microbial from soils with low pH in our work, possibly indicating greater nitrogen fixation activity in lower pH conditions.

In terms of overall functional genes, functional gene analyses of KEGG pathways showed clear differences in rice soil microbial functions at different soil pH and salinity (Figures 4A,B) as well as carbohydrate-active enzymes (Figures 4C,D). These results implied that the range of soil microbial functions is presumably influenced by soil pH and salinity.

The functional composition of microbial communities in natural environments such as soil depends strongly on environmental factors (Nelson et al., 2016; Gibbons, 2017; Louca et al., 2017). Microorganisms that live in similar habitats perform similar ecological functions, but the composition of the microbial species that perform those functions may be quite different (Gibbons, 2017). Therefore, revealing the functional profile of microbial communities is particularly important, as is revealing which microorganisms reside in the environment (Nelson et al., 2016; Gibbons, 2017; Louca et al., 2017). Microorganisms with diverse functional groups maintain soil functions, and functional complementarity exists within specific functional groups of species. A decline in any one group of species has little effect on soil ecosystem function since other bacteria and fungus can assume their functions (Delgado-Baquerizo et al., 2017). Our results demonstrated that the functional structure of soil microorganisms was maintained by different rhizosphere microbiome assembly (Figures 4B,D).

## 5 Conclusion

In conclusion, this study provides valuable insights into the rhizosphere microbial communities of different rice cultivars grown in saline-alkaline soils in northeastern China. The results highlight the significant influence of soil salinity-alkalinity and rice variety on the composition of rhizosphere microbiomes, which in turn affects rice yield and grain quality. The distinct microbial communities associated with Jida177 and Tongxi933 suggest that the rhizosphere microbiome plays a critical role in enhancing salinity-alkalinity tolerance. This research opens new avenues for the selection and potential reintroduction of specific rhizosphere bacteria to improve microbial diversity and rice productivity under saline-alkali stress. To realize the full potential of these microbiomes, further investigation of the microbial community structure and its mechanisms of action is essential. Future metagenomic and metaproteomic studies will be crucial to identify microbial consortia that can be used as biofertilizers to promote sustainable rice production, thus providing an environmentally friendly approach to saline-alkali soil management.

## Data Availability

The data presented in the study are deposited in the NCBI repository under BioProject number PRJNA1241858 and accession numbers SRR32855518–SRR32855529.

## Footnotes

^1^ www.illumina.com

^2^ http://www.ncbi.nlm.nih.gov/Taxonomy/taxonomyhome.html/index.cgi?chapter=tgencodes#SG1
